# Differences in malaria and haematocrit presentation in children living in different settings, North West Region, Cameroon

**Published:** 2021-06-01

**Authors:** Ebanga Echi J. Eyong, Hyloson Nkwengang, Laurentine Sumo

**Affiliations:** 1 Department of Biological Sciences, Faculty of Science, University of Bamenda, P.O. Box 39, Bambili, North West Region, Cameroon.

## Abstract

**Background:**

Malaria continues to be a major cause of morbidity and mortality in Cameroon. With all efforts being made to eliminate malaria, it is imperative to describe the epidemiology of the disease in different parts of the country in order to inform control policies. This study aimed to present the differences in the prevalence and intensity of malaria and the anaemic status of children living in different areas of the North West region of Cameroon.

**Materials and Methods:**

This study was carried out from April 2016-July 2017. Blood samples were collected from children via finger pricking. Stained thick and thin blood films were examined through microscopy (x100) to detect the presence of parasites and to estimate the geometric mean parasite density (GMPD). Packed cell volume (PCV) values were determined by micro-centrifugation. Data was analysed using SPSS to determine proportions and test for significance levels between these.

**Results:**

Overall prevalence of malaria was 45.3%. Awing and Obang recorded the highest prevalence while Mankon and Nkwen recorded the lowest (p=0.01). The GMPD of infection was highly heterogeneous between the different localities (p=0.03). Age significantly affected the prevalence of malaria (p=0.02). Sex did not affect the prevalence nor the GMPD of malaria infection (p>0.05). Overall mean PCV value was 32.9±3.9. Localities in urban settings recorded the highest mean PCV values compared to those in rural settings (p=0.68). Sex and age did not affect mean PCV values (p>0.05).

**Conclusion:**

Malaria still remains a major problem in the North West region of Cameroon. Malaria control interventions should therefore be based on evident spatial and temporal heterogeneity of *Plasmodium* species in a particular area so as not to waste resources that would only be of limited effectiveness and value to the populations at risk.

## Introduction

Malaria is a disease of great public health concern, especially in tropical and sub-tropical parts of the world, where some 3 billion people in more than 100 countries live at risk [1]. Cameroon, a malaria-endemic country in Central Africa has witnessed a decline in the incidence of malaria, largely attributed to the relentless effort by its government to ensure universal coverage with insecticide treated bednets [2]. Despite these efforts, the disease still remains one of great concern, with over 90% of Cameroonians at risk of malaria infection, and ~ 41% witnessing at least one episode of malaria each year [3]. Cameroon, located between latitudes 2°N and 13°N, is located between West and Central Africa and is often described as ‘Africa in miniature’ because of the diverse natural environment spread across the country in its ten regions [4].

Cameroon is blessed with the greatest biodiversity in Africa. The highland plateau of the West and North West regions constitutes the savanna zone. However, continuous human activities e.g. changing agricultural practices, deforestation, etc., have resulted in changes in landscape ecology and climatological parameters. These changes have not only impacted the biogeography of the region but also favour the transmission of both new and existing infectious diseases. Several studies carried out in different parts of the country show differences in the prevalence of malaria [2,5,6]. However, no such study has been carried out in the North West Region of Cameroon.

According to WHO [7], Cameroon, is amongst the 11 countries (Burkina Faso, Cameroon, Democratic Republic of the Congo, Ghana, India, Mali, Mozambique, Niger, Nigeria, Uganda and United Republic of Tanzania) ranked as having the highest burden of malaria. As Cameroon pursues its goal to reduce malaria-associated morbidity and mortality and eventually eliminate it from the country, there is an urgent need for empirical studies to establish an evidence-based distribution to inform control policies. Such studies will provide strategic information to drive impact.

Anaemia is a known complication of malaria and other infections. It has a profound effect on the quality of life of people by inducing such symptoms as loss of stamina, rapid heart rate and shortness of breath [8]. Anaemia has been reported to be a major cause of death in people suffering from malaria [9]. Although studies [10] exist on the anaemic situation of people with malaria in other parts of Cameroon, none has focused on the North West Region of Cameroon. Recently, bednets were distributed in some localities of the region but malaria still remains one of the major reasons why people visit hospitals in this region. Against this background, our study was designed to determine the prevalence and intensity of malaria infection in different localities, with respect to age and sex and levels of anaemia in the study population.

## Materials and Methods

### Study area

This study was carried out in six localities (Mankon, Nkwen, Batibo, Bambui, Awing and Obang) in the North West Region of Cameroon, characterised by savanna, with two distinct seasons; the dry season from November to February and the rainy season which is wet and cool which spans from March to October. The region is made up of many towns and villages, which can be categorised as urban, semi-urban or rural depending on characteristics that include land use, household density, types of housing, access to public transport, access to utility services and access to social services.

### Study design and Selection criteria

A cross-sectional study was carried out between April 2016 and July 2017. Children, aged 4-16 years, of both sexes, were used as proxy to estimate the parasitological indices and haematocrit values. Children selected were those whom a malaria test and haematocrit tests had been requested for by a medical practitioner. Children were selected because they are a high-risk group for malaria and constitute the majority of hospital consultations in many health facilities throughout the region.

### Administrative and ethical considerations

Authorisation for the study was obtained from the North West Regional Delegations of Public Health and the chief medical officer from the respective clinics. The chiefs and their council members in each locality were visited and sensitised on the benefits of the study. One clinic or hospital in each locality was used for sample collection. Participation for blood sample collection was voluntary and parents/ legal guardians had to fill and sign an informed consent form, which explained the benefits of the study (i.e., infected children would receive treatment).

### Sample size estimation

The sample size for this study was calculated based on a prevalence obtained by Mbenda *et al.* [3] using the Cochran formula [11] with formula:

$$n=Z^{2}pq/e^{2}$$

where Z = standard number deviate (1.96), n = desired sample size, p = prevalence of malaria, 90%, q =1-q (proportion in the population that does not have the characteristics being measured), e = desired level of precision, n = (1.96)^2^(0.9)(0.1)/(0.05)^2^, so n = 138.29 x 6 = 830.

To get the number of participants in the different localities, we multiplied by the 6 since we worked in 6 localities. However, in some localities we could not reach the required sample size because many potential participants were not available due to socio-political unrest or had moved into other towns.

### Blood collection, preparation and staining of blood smears

Prior to blood sample collection, demographic information such as the age, sex, and place of residence were recorded. Using sterile disposable lancets, finger pricks were performed. Thick and thin blood films were prepared using the method described by Cheesbrough [12]. The code number of each individual was written on the slide and the blood films were allowed to air dry protected from dust and flies. In the laboratory, the thin films were fixed with 100% methanol for one minute and both thick and thin blood films were stained with 5% Giemsa stain solution for 30 min [12].

### Detection and estimation of parasitaemia of Plasmodium species

The slides were read under x100 (oil immersion) objective by an experienced microscopist. A second experienced microscopist, blinded to the first reading, read all thick smears and any discrepancies (positive vs. negative; results that did not match each other; >25% difference in parasite density) were resolved by a third microscopist. Parasite densities were determined from thick blood smears by counting the number of asexual parasites or sexual parasites per 200 white blood cell count (WBC) and converted to number of parasites/μl blood assuming a standard WBC of 8,000/μl. A smear was considered negative if no parasites were seen after review of 100 high-powered fields.

### Determination of packed cell volume (PCV)

Haematocrit values were determined by micro-centrifugation of blood samples in heparinised capillary tubes. PCV values of ≤20% were diagnostic of severe anaemia, values of 21-29.9% of mild anaemia and values ≥30% were considered to be normal values [12].

### Data analysis

Data was entered in Excel and analysed using statistical software SPSS version 20. The Chi square test was used to assess significance levels for differences between proportions. Descriptive statistical analyses were performed to compute the mean and standard deviations of parasite counts and haematocrit values in the general study group, and in both males and females. The prevalence of malaria was determined for localities in the different settings. Intensities were obtained by calculating geometric means and expressed as geometric mean parasite density (GMPD) per locality. Simple Chi square analysis was performed to compare different proportions. The Kruskal-Wallis test was used to compare parasite densities in the different localities. All tests were performed at the 5% significance level.

## Results

### Demographics of the study population

Across all six localities, the total number of children sampled was 876. 42.6% (n=373) of these were males and 57.4% (n=503) were females. The proportion of pupils per age group were as follows: 27.3% (n=239) for the age group 4-8 years; 57.3% (n=502) for the age group 9-12 years and 15.4% (n=135) for the age group 13-16 years. The mean age (±SD) of the study population was 9.84 2.45 years. Table 1 shows the number of pupils examined per locality.

**Table 1:** Demographic characteristics of the study population.

Parameter		#Examined	%
Locality	Mankon	146	16.6
	Nkwen	107	12.2
	Batibo	75	8.6
	Bambui	188	21.5
	Awing	159	18.2
	Obang	201	22.9
Sex	Male	373	42.6
	Female	503	57.4
Age (yrs)	4-8	239	27.3
	9-12	502	57.3
	13-16	135	15.4

### Prevalence and density of malaria with respect to localities in different settings

Out of the 876 children sampled, 397 (45.3%) were positive for malaria recording a parasite density of 690.89 (40-48,000 parasites/μl of blood); see Table 2. The highest (70.6%, n=142) prevalence of malaria was recorded in Obang, a rural area while the lowest prevalence was recorded in Mankon (12.3%, n=18), an urban area. The differences in prevalence of malaria across the different localities was significant (P=0.01). The highest GMPD of 839.42 (40-29520 parasites/μl of blood) was recorded in Awing, a rural area while the lowest GMPD of 496.14 (120-4200 parasites/μl of blood) was recorded in Nkwen, and again this difference was significant (P=0.03).

**Table 2:** Prevalence and intensity of malaria infection in different localities.

		Parasitological data		
Locality	Setting	#Examined	#Positive* (%)	GMPD** (Range)
Mankon	Urban	146	18 (12.3)	522.53 (120-12000)
Nkwen	Urban	107	19 (17.8)	496.14 (120-4200)
Batibo	Semi-urban	75	31 (41.3)	650.29 (120-48000)
Bambui	Semi-urban	188	88 (46.8)	700.39 (120-16240)
Awing	Rural	159	99 (62.3)	839.42 (40-29520)
Obang	Rural	201	142 (70.6)	656.34 (40-480000)
Total		876	397 (45.3)	690.89 (40-48000)

*P=0.01; **GMPD = Geometric Mean Parasite Density, Range shows lowest and highest values, P=0.03.

### Prevalence and density of malaria infection in localities of different settings with respect to sex

Females recorded a higher prevalence 48.7% (n=245) of malaria than males (40.7% (n=152)), although this difference was not significant (P=0.21); see Table 3. Parasite density in different localities with respect to sex is shown in Table 3. There was no significant difference in GMPD between sexes in the different localities (P=0.51).

**Table 3:** Prevalence and intensity of malaria infection by sex and locality.

		Male*	
Locality	#Examined	#Positive (%)	GMPD** (Range)
Mankon	83	9 (10.8)	348.63 (120-3360)
Nkwen	31	4 (12.9)	491.95 (200-1760)
Batibo	37	16 (43.2)	585.43 (120-280)
Bambui	76	30 (39.5)	862.01 (160-5160)
Awing	72	42 (58.3)	927.58 (40-29520)
Obang	74	51 (68.9)	638.14 (120-12000)
Overall	373	152 (40.8)	713.04 (40-29520)
		**Female**	
Mankon	63	9 (14.3)	783.15 (120-12000)
Nkwen	76	15 (19.3)	497.26 (120-14200)
Batibo	38	15 (39.5)	727.87 (160-16000)
Bambui	112	58 (51.8)	629.07 (120-16240)
Awing	87	57 (65.5)	779.60 (40-20000)
Obang	127	91 (71.6)	666.76 (120-48000)
Total	503	245 (48.7)	677.49 (40-48000)

*Prevalence between sexes, P=0.21; Intensity between sexes, P=0.51. **GMPD=Geometric Mean Parasite Density; Range shows lowest and highest values.

### Prevalence and density of malaria infection in the different localities with respect to age

The age group 9-12 years recorded the highest prevalence 56.9% (n=226) of malaria while the age group 13-16 years recorded the lowest prevalence 15.1% (n=60). Differences in prevalence of malaria was significant with respect to age across the different localities (P=0.02, Table 4). Parasite densities in the different age groups are shown in Table 4; the difference between age groups across the different localities was not significant (P=0.83).

**Table 4:** Prevalence and density of malaria according to age and locality.

		Age group (years)					
Locality	#Examined	4-8		9-12		13-16	
		N +ve* (%)	GMPD** (range)	N +ve (%)	GMPD (range)	N +ve (%)	GMPD (range)
Mankon	146	5 (3.4)	1006.38 (200-12000)	10 (6.9)	293.58 (120-1400)	3 (2.1)	1197.63 (800-2440)
Nkwen	107	3 (2.8)	402.62 (200-4200)	13 12.2)	473.64 (120-1760)	2 (2.8)	230.76 (160-480)
Batibo	75	13 (17.3)	662.67 (200-16000)	18 (24.0)	650.67 (120-2880)	0 (0)	0 (0)
Bambui	188	21 (23.9)	676.81 (120-8960)	53 (60.2)	641.21 (120-5160)	14 (15.9)	1029.91 (160-16240)
Awing	159	21 (13.2)	779.75 (120-20000)	60 (37.7)	1089.20 (40-20000)	18 (11.3)	791.99 (40-29520)
Obang	201	48 (33.8)	696.68 (120-48000)	72 (50.7)	675.36 (120-12000)	22 (15.5)	524.80 (120-2360)
Total	876	111 (28.0)	766.12 (120-48000)	226 (56.9)	655.64 (40-20000)	60 (15.1)	695.04 (40-29520)

*Prevalence of malaria between age groups, P=0.02; **Intensity of malaria between age groups, P=0.83; GMPD=Geometric Mean Parasite Density; Range gives the lowest and highest values.

### Haematocrit profile in the study population

Overall, a mean PCV value of 32.97±3.94 was recorded (n=872); see Table 5. Two localities in the urban settings recorded the highest mean PCV values of 34.82±3.93 and 34.63±3.95 while the two localities in the rural setting recorded the lowest mean PCV values (31.52±3.88 and 30.51±5.38). There was no significant difference (P=0.681) in mean PCV values across the localities.

**Table 5:** Mean ± Standard Deviation (SD) packed cell volume (PCV) values of the study population according to locality, sex and age.

Characteristic	#Examined	Mean PCV±SD	P value
**Locality**			
Mankon	145	34.82 ± 3.93	0.681
Nkwen	106	34.63 ± 3.95	
Batibo	75	33.72 ± 3.15	
Bambui	186	32.62 ± 3.36	
Awing	159	31.52 ± 3.88	
Obang	201	30.51 ± 5.38	
**Sex**			
Male	371	33.09 ± 3.93	0.532
Female	501	32.81 ± 3.94	
**Age group (years)**			
4-8	236	34.44 ± 3.35	0.831
9-12	502	31.37 ± 5.37	
13-16	134	33.34 ± 3.11	
Total	872	32.97 ± 3.94	

Males recorded a higher mean PCV (33.09±3.93) than females (32.81±3.94), although there was no significant difference (P=0.532) in between sexes. The highest mean PCV value (34.44±3.35) was recorded in the age group 4-8 years while lowest mean PCV value (31.37±3.11) was recorded in the age group 9-12 years; no significant difference (P=0.831) was recorded in the mean PCV value with respect to age groups. The missing 0.5% accounts for 4 PCV tubes which got broken in the course of transportation from the field to the laboratory.

## Discussion

The results of this study show that the prevalence of malaria in different localities in the North West region exhibits a highly heterogeneous profile. The overall prevalence of 45.3% recorded in this region suggests that malaria still remains a major problem in this part of Cameroon. This high prevalence of malaria recorded, despite all the efforts made by the government to curb the disease burden, is probably due to the fact that many of the inhabitants in this region do not use the long-lasting insecticide-treated bednets (LLINs) that were distributed to different homes, coupled to other factors which favour the occurrence of malaria such as temperature, rainfall, relative humidity, presence of breeding sites and topography [7,13]. The use of LLINs is one of the cheap and easy to use methods recommended to prevent malaria. One study carried out by Ntonifor *et al.* [14], in some localities in the North West region revealed that nets handed out to inhabitants in the North West region were used for fishing, nursing of seeds, and football nets with the reasons being that the use of these nets cause a lot of heat and leaves a feeling of suffocation when slept under.

Amongst the localities sampled, the prevalence of malaria was higher in the rural areas than in urban and semi-urban areas. Obang and Awing recorded the highest prevalence, which is probably due to the fact that children living in these localities are more exposed to malaria transmission as a result of higher vector density, poor quality housing, poor drainage systems and difficulties in assessing healthcare facilities [15,16]. Although socio-economic factors were not considered in this study, we observed that most of the houses in the rural areas were built from mud and most of these houses have holes and crevices in the walls with no form of screens either on the doors or windows. Human-vector contact is influenced to a great extent by housing type, housing and roofing material, house location, gradient, surrounding drainage and cleanliness of immediate environment [17-19]. Some studies have shown that when compared with urban areas, mothers living in rural or semi-urban communities have lower vaccination coverage, poorer physical access to health services and lower use of LLINs, and lack of screens on doors and windows. Hence, there is always increased transmission of vector-borne diseases, such as malaria, in such areas [20].

The prevalence of malaria was higher in females than in males, although the difference was not significant. This disagrees with other studies that have reported higher prevalence rates of malaria in males [21]. There is evidence that females have better immunity to malaria and a variety of other parasitic diseases than males and this has been associated with hormonal or genetic factors or both [22].

The mean intensities of malaria infection varied greatly between the different localities and the difference was significant. The great heterogeneity observed in the mean intensities of malaria infection is probably due to the fact that parasite density distribution is not uniform among pupils in the different localities. Generally, younger children (4-8 years) were found to have higher parasite counts than their older school mates, although none presented with clinical signs or symptoms. It is known that immunity to malaria infection builds up with multiple exposures to the infection. Thus, as children get older they have probably had several attacks thereby developing some partial immunity which aids in reducing the parasite load and suppressing clinical manifestations of the disease.

We found out that urban localities recorded higher mean PCV values than rural localities, although the difference was not significant. The higher mean PCV values recorded in children in urban areas coupled with a low prevalence and GMPD/ μl of blood probably reflects the impact of less malaria transmission intensity when compared to the rural areas. The findings of this study differ greatly from that of Sumbele *et al.* [10], who found anaemia to be higher in rural areas. Ignorance, poverty, and gender bias also contribute to high prevalence of anaemia [23] and these factors are rife in rural areas in the North West region of Cameroon. Mean PCV values were lower in children in the age group 9-12 years than in the other age groups. This was probably due to the fact that this age group recorded the highest prevalence of malaria. Although none of these children were anaemic, it is a well-known fact that malaria parasites cause the destruction of red blood cells, which probably accounts for the low mean PCV values recorded in these children. Males had higher PCV values than females, although the difference was not significant. This is probably because males have a greater muscle mass than females. Although malaria parasites feed on haemoglobin of red blood cells leading to lysis of these cells and consequently a lower haematocrit [24] there are other factors such as hookworm infection or iron-deficiency anaemia which contribute to anaemia. Although these factors were not investigated in the present study, there is a possibility that they might have contributed to the low PCV values recorded in females.

## Conclusions

This study showed that the prevalence of malaria in localities of different settings in the North West region of Cameroon shows a heterogeneous pattern. Localities in rural areas (Awing and Obang) recorded the highest (70.7%, 62.3%, respectively) prevalence while those in the urban areas (Mankon, Nkwen) recorded lowest (12.33%, 17.8%, respectively). The highest mean intensity of malaria (839.42 parasites/μl blood) was recorded in Awing whereas Nkwen recorded the lowest (496.14 parasites/μl blood). Malaria prevalence in children aged 9-12 years was higher than in other age cohorts. Generally, younger children (4-8 years) were found to have higher parasite counts than those in other age groups, although none presented with clinical signs or symptoms. Higher mean PCV values were recorded in the urban localities while lower mean values were recorded in the rural localities. Assessing variations in malaria prevalence/ intensity can be useful when allocating (limited) resources to malaria management and control. Governments, through health facilities, should improve sensitisation and education on malaria in order to reduce ignorance and lack of awareness which makes people neglect their health. Also, people should be educated on food substances that can improve their haematocrit values.
